# Extramedullary Hematopoiesis in Gastric Mucosa

**DOI:** 10.3390/diagnostics15101219

**Published:** 2025-05-12

**Authors:** Matilda Djolai, Jovana Baljak, Tanja Lakić, Jelena Ilić Sabo, Željka Panić, Aleksandra Ilić, Vladimir Vračarić, Sandra Trivunić Dajko

**Affiliations:** 1 Center for Pathology and Histology, UKCV Novi, 21000 Novi Sad, Serbia; matilda.djolai@mf.uns.ac.rs (M.D.); tanja.lakic@mf.uns.ac.rs (T.L.); jelena.ilic-sabo@mf.uns.ac.rs (J.I.S.); aleksandra.m.ilic@mf.uns.ac.rs (A.I.); sandra.trivunic-dajko@mf.uns.ac.rs (S.T.D.); 2 Department of Histology and Embryology, Faculty of Medicine, University of Novi Sad, 21000 Novi Sad, Serbia; zeljka.panic@mf.uns.ac.rs; 3 Department of Pathology, Faculty of Medicine, University of Novi Sad, 21000 Novi Sad, Serbia; 4 Emergency Center of UKCV, 21000 Novi Sad, Serbia; vracaricv@gmail.com

**Keywords:** extramedullary hematopoiesis, stomach, myelofibrosis

## Abstract

In this paper, pathohistological images of extramedullary hematopoiesis (EMH) in stomach mucosa in a 68-year-old female patient with treated osteomyelofibrosis are presented. The digestive system is a potential but uncommon site of EMH, with the gastric mucosa being particularly rare. According to the available literature, only 12 cases have been described.

**Figure 1:**
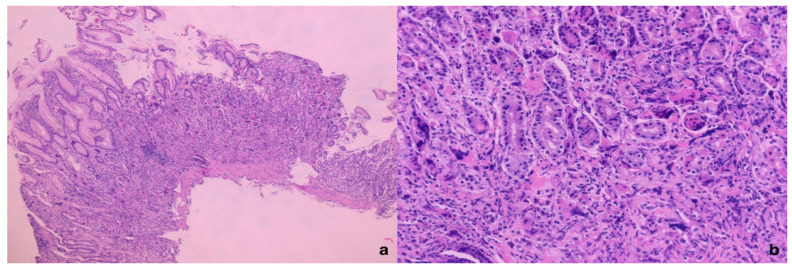
The microphotography of the gastric mucosa with extramedullary hematopoiesis, H&E×50 (**a**) and H&E×200 (**b**). A 68-year-old female patient with previously diagnosed and treated osteomyelofibrosis consulted, due to gastrointestinal complaints, a gastroenterologist who indicated gastroscopy. In the gastroscopic findings, a “polypoid” formation was seen in the subcardiac region, which was completely removed and forwarded for pathohistological analysis. After routine pathohistological processing, the pathohistology of the “polypoid” lesion is described as follows: reduced glandular structures, fibrosis in the lamina propria, abundant infiltrate of lymphocytes, plasma cells and, also, in the lower and middle thirds of the lamina propria, giant cells with irregular rosette-like nuclei. The giant cells did not have the usual cytological features of reactive giant cells or macrophages, hence further standard immunohistochemical analysis was performed with: Factor VIII, CD61, and CD31, AE1/AE3, CD68, CD117, CD34, Desmin, Estrogen, and Mammaglobin.

**Figure 2:**
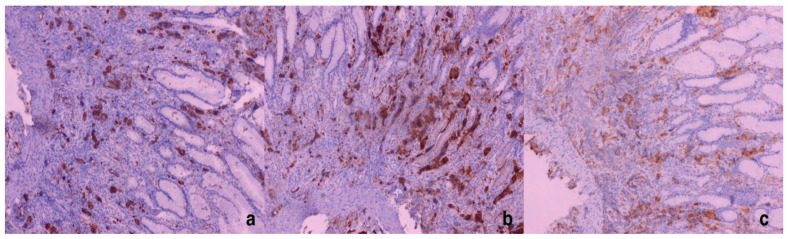
Giant cells—megakaryocytes stained immunohistochemically with Factor VIII (**a**), CD61 (**b**), and CD31 (**c**); ×100.

**Figure 3:**
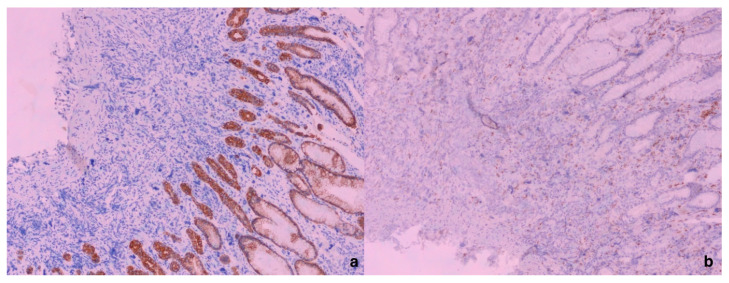
Giant cells—megakaryocytes immunohistochemically negative for AE1/AE3 (**a**) with positive internal immunohistochemical control (AE1/AE3 positivity in surface and foveolar epithelium) (×100). In (**b**), it is shown that giant cells, megakaryocytes, were negative for CD68, in comparison with CD68-positive macrophages (×100). Based on the performed immunohistochemical analysis and giant cell positivity for Factor VIII, CD61, and CD31, the megakaryocytic lineage of the gastric mucosa was confirmed. Because of this giant-cell immunohistochemical positivity, we suspected EMH in the gastric mucosa and, in the meantime, it was revealed that the patient had been treated for osteomyelofibrosis.

**Figure 4:**
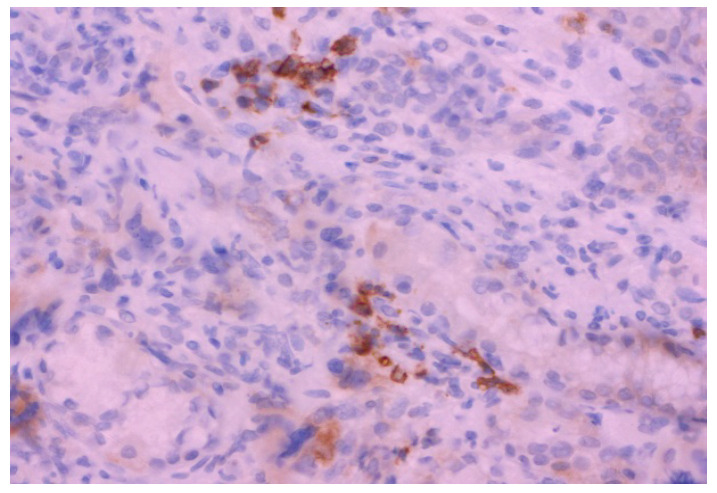
The microphotography of CD 71-positive cells in the stomach mucosa (erythroid lineage); ×400. In order to establish the diagnosis of EMH, in addition to the megakaryocytic lineage, erythroid lineage must also be present; hence, further immunohistochemical analysis with the CD71 antibody was performed. Immunohistochemically, CD 71-positive cells were present in the same histological sections, which proves erythroid lineage. The digestive system is a potential but uncommon site for EMH, with the gastric mucosa being particularly rare. According to the available literature, only 12 cases have been reported [1]. The EMH of the gastric mucosa in this case had a clinical and pathohistological presentation like most cases in the literature available to us [1]. The presence of giant cells in the mucosa of the digestive system is always a differential diagnostic dilemma in relation to giant tumor cells [2]. According to data from the literature, multinucleated cells can be found in the upper parts of the digestive tract as part of infectious diseases (usually of the viral type), inflammatory diseases, radiotherapy, and tumors, but benign megakaryocytes can also occur as part of myelofibrosis (megakaryocyte nuclei are larger than in reactive multinucleated stromal cells or giant cells) [2]. The proliferation of cells within the EMH in atypical places (newly formed and accumulated cells) clinically most often resemble a tumor, while symptoms depend on the localization and size of the newly formed tumefact and/or the physical symptoms of compression [1,3]. In order to establish a diagnosis of EMH, especially in non-specific places such as the digestive tract, insight into the patient’s complete medical history is very helpful [4].

## Data Availability

The original contributions presented in this study are included in the article. Further inquiries can be directed to the corresponding author.
